# Selections from the French

**Published:** 1888-06

**Authors:** S. Pozzi

**Affiliations:** Fellow-Surgeon to Lourcine Pascal Hospital


### SELECTIONS FROM THE FRENCH.

### ANTISEPTICS IN GYNECOLOGY.

BY DR. S. POZZI, FELLOW-SURGEON TO LOURCINE PASCAL HOSPITAL.

Translated by F. J. Arbeely, A. B., M. D., Atlanta, Ga.

CONCLUDED

### I.

Before I close my remarks on the use of antiseptic method to the external genito-organs, vagina and os-uteri, let me add a few words concerning the manner in which it is better employed. I mean to say the continuous irrigation. You not only disinfect these organs by this means, but you will remove at the same time whatever objectionable matter that may be left there which might tend to defeat the object of an antiseptic operation. A special speculum or simply an assistant holding the nozzle properly, with his hand resting on the pubis, will aid in accomplishing this purpose. The solution which I generally use is the carbolic acid, 1 to 1.000 of 35 to 40 degrees temperature. If a small stream of the liquid was thrown in more or less actively, it will run immediately over the operative field, and accomplish two objects: first, it washes out the blood without the aid of a sponge or any similar article; and second, it maintains the wounded organs bathed with the antiseptic, protecting them from the germs in the atmosphere during the operation. For this reason I think that irrigation here is better than the use of the spray, as a rule. I irrigate invariably whenever I operate on these parts of the body. I am favorably impressed with irrigation, and cannot recommend it too highly to my colleagues, who, if I am not mistaken, do not take any interest in it as yet.

II.

Now I will speak about those important precautions, which must be observed in antiseptic methods in laparotomy. There is quite a prejudical spirit against antiseptic surgery, even among those operators who occupy the highest rank in the profession—Lawson Tait, Keith and Bantock, for instance. While they admit the remarkable success they had when this method was adopted in their practice, they declare that it is not only useless, but dangerous to the patient. Now, does not such action seem like dodging the true fact? and it simply confirms peremptorily the views of these operators, who believe in taking the greatest care and minute precautions which I am about to lay before you.

The seemingly great contradiction in this question is really very trifling. To satisfy yourselves of the true fact, look into the practice of these eminent operators, and examine, if you please, all the particulars. There you will certainly see their actual views, if not exactly in accordance with the antiseptic, they are, to a great measure, aseptics, observing the rules of antisepsis. The actual cautery which they often apply to the raw surfaces of pedicles, or to divide an adhesion, etc., is it not in some sense similar to the use of antiseptics in destroying the germs or modifying their field of culture? The truth is, that the action is one, but the word only is not used. These parenthetical remarks being given, let me return to my subject.

A.—The operator and assistants must be perfectly clean. Should any one have been in the dissecting or necropsy room, touching or mingling with anatomical subjects or septic matter previously, he must wait twenty-four hours at least before operating or assisting in the operation; otherwise a sublimate or vapor bath, followed by soap and water scrubbing, must be taken first. They, all operators and assistants, must wear long and clean linen coats or aprons; care taken not to touch a thing which is not disinfected, and in case of necessity, a clean pair of gloves worn until the operation begins.

B.—The patient should take a sublimate or soap and water bath the evening before, vaginal injections with sublimate solution 1 to 2,000; each one, followed by a tampon of iodoform, must be practiced several days before the proposed operation; bowels emptied by a large enema the same morning or the evening before; urine drawn off ,skin invariably shaved, abdomen scrubbed with brush, soap and water, either, then with sublimate solution 1 to 1.000; special care must be taken in cleaning the umbilical wrinkles; finally a compress saturated with the sublimate is adjusted to the abdomen in such manner as to allow the liquid to run in and get mixed with others during the operation.

C.—Location; choose the room which is located as far as possible from the other departments of the hospital, especially if there is septic matter or suppurating wounds, or water-closets which frequently are the sources of infection; walls, ceiling and floor must be closely made so as to be easily washed with plenty of water, the corners round and accessible so as to be well cleaned, furniture easily removed, seats, tables and shelves all constructed of enameled metal, varnished or exclusively glass.

A general cleaning up should follow each operation; a rubber hose, armed with a nozzle, attached to a pump in the other end, will throw the water in a sufficient force to dislodge and remove whatever septic matter or other impurity might be found there. If the operation has to be made in a private house, two days at least should be allowed for preparations; furniture removed from the room, walls white-washed, floor and all the wood-work properly cleaned with sponges soaked in carbolic acid solution 50 to 1.000; to be more particular, a sulphurous acid gas should be let out in the room all day alone.

The temperature should be carefully watched and maintained between 20 and 30 degrees, to keep the patient from taking cold, and the atmosphere from getting excessively dry, as either one is very dangerous to the exposed viscera. A spray's current of carbolic acid solution should be directed to the middle and all round in the room, but not to the operative field, as was practiced in the day of Listerism, and even now-a-days by some ovariotomists, because when such current is directed toward the patient it is certainly injurious, as it does not only chill the exposed organs, but will irritate the peritoneum also, and might cause intoxication; hence the main point to be observed in the spraying is, saturate the air thoroughly with the steam first; if the operation is prolonged, repetition of the same would be necessary then.

D.—*Instruments.* The instruments which had been already cleaned and kept 5 or 10 minutes in boiling water after a previous operation, should be subjected to 110 or 120 degrees of heat for half an hour again, then immersed in carbolic acid solution, 50 to 1.000, immediately before the operation, but you must remember that an instrument which has gone through this process must be sharpened again.

To be sure that they are perfectly clean you had better use your own instruments. A dull, disinfected instrument is by far better than a sharp and suspected one in the success of the operation.

For the same reason I renounce the use of common sponges, as it is a very difficult matter to find a new and perfectly clean one. To comprehend this statement look at the amount of care required to render them properly fit for use in their preparation, especially when they are old or have been used before. You must not be surprised at my suggestion, if I say that this important fact often is indifferent in hospital practice. Another objection to sponges which has led me to substitute them by the compressed antiseptic-sponges, which are generally used by Professor Billroth, is that they are either too hard or too soft and infirm, consequently inconvenient to both operator and patient.

The compressed antiseptic-sponges are prepared as follows:

Take a sheet of gauze, double it up until you form a square 30 centimes thick, which is composed of eight layers, hem the edges evenly all around, boil in carbolic acid solution, 50 to 1,000, or in the sublimate, 1 to 1,000, at least two hours, then keep in a similar solution, which you must renew once a week. Previous to an operation they must be washed quickly with sublimate solution, diluted with the same amount of sterilized water, then squeezed well. Made in this way, the compressed sponges are powerfully absorbent, thoroughly aseptic, soft and pliable agents, accessible to any shape or size, and very accommodating to the hands or fingers in cavities or interstices; in fact, they offer much more superior advantages than the common sponge does; they can be cleaned and used more than once during the period of an operation, except when they become soiled with septic matter; but at the end of each operation all should be thrown away, as they are cheap and easily to be made.

Let me call your attention for a moment to those antiseptic methods which really form a part of the operation itself. I will mention only here those which come under this head of my subject.

A.—There is a great benefit to be derived from washing the peritoneal cavity with sterilized warm water (to which I frequently add 6-1,000 chloride of sodium). Lawson Tait speaks very highly of its advantage, especially in those cases where the serous membranes have been soiled with an infectious liquid or irritant agent during the operation; but good care should be taken not to overdo the thing. There is a good deal more danger in leaving the least particle of pus or septic matter than to leave several small clots of blood in the cavity; besides this, general washing out will assist the reaction from the severe depression of the shock.

B.—Touching the raw surfaces of a wound, pedicles or adhesions with an antiseptic, as with strong carbolic acid solution, tincture of iodine, iodoform or the actual cautery. The use of the latter is very much admired by Baker-Brown and extensively practiced in England and Germany, but rarely in France. For my part, I use it frequently whenever I see a suspected surface (in certain forms of salpingotomy, for instance), not as a hemostatic to arrest a hemorrhage, for which it is regarded as excellent, but simply as an antiseptic, for the thermo-cautery of Paquelin has done away with the use of Brown's hot iron.

As I am about to close my subject, let me make a few remarks about the various methods in which you can prepare and preserve these particular agents in use for ligatures, sutures, etc.

*Silk.*—Among the best, strongest and most tenacious agents used is the braided silk. Roll the silk thread on a glass quill, boil in carbolic solution, 50 to 1,000, two hours, then transfer to a similar solution, which must be renewed once a week until it is used.

It is best not to prepare a big supply at once, because such strong solution will render it indurable and brittle.

Hegar prepares it with iodoform after this manner:

Immerse the silk first in iodoform colodion (20 grams of iodoform to 200 grams of ether), leave it in 24 hours, take out and let it dry, roll it on a glass quill with iodoform dust on, then keep in a well-closed bottle until it is used.

Other surgeons prepare it with sublimate solution 1 to 1,000.

I prefer myself the carbolized silk, where I am obliged to make many sutures, especially when I have to leave them in the abdominal cavity, because I feel then that I have run no risk of intoxication, which occurs sometimes to weakly and susceptible persons to the sublimate effect.

*Cat-gut.*—I always have the best results in my operations whenever I use cat-gut, which is prepared with oil of juniper berries (oleum ligni juniperi). Cat-gut is immersed three hours in sublimate solution 1-1,000. It is then transferred to and kept eight days at least in oil of juniper berries; then it is preserved in pure alcohol. Shortly before the beginning of the operation the cat-gut is returned into a similar solution of the sublimate which swells it and renders it flexible and better fit for use.

Martin, however, prepares it a little different. He rolls the cat-gut on a glass quill, immerses and keeps for two hours in the sublimate solution, wipes it out with a clean towel, then keeps it in a mixture of two parts of alcohol and one part of oleum juniperi for six days before operating, the amount required only taken out and placed in a basinful of some sort of an antiseptic.

Cat-gut which is prepared with oil of juniper is very extensively used. It is much superior to that which is disinfected and prepared with carbolized oil; its tenacity and flexibility are remarkable, very convenient and safe when the operator has to leave the sutures in a wound or cavity, as they will be dissolved and absorbed sooner or later, according to the size and time, which must be judged by the practical operator. Schrœder, then Martin, were the first observers of these special superior qualities of the cat-gut. They both had a remarkable success in their use.

*Elastic rubber bandages, tubes, and drainage tubes.*—The usual method to disinfect these agents by the immersion in carbolic acid or sublimate solution is impracticable here, as it is not sufficient to destroy the germ's eggs. To be subjected to 110° of heat is out of the question, because they will soon be liquefied. Therefore, to accomplish this object safely, Force advises the following process: Stimulate the egg's development first by immersing and keeping these agents in warm water of thirty-five degrees temperature for nearly six days, until the eggs are hatched, then destroy the germs by the sublimate or carbolic acid solution, 50 to 1,000; renew this solution every couple of days for a fortnight. The result is that you will have a perfectly safe and disinfected agent.

